# Theranostic Nanoplatforms for Alzheimer’s Disease: A Critical Analysis of Conceptual Contradictions

**DOI:** 10.3390/ijms27083560

**Published:** 2026-04-16

**Authors:** Yana Zorkina, Olga Abramova, Eugene Zubkov, Olga Gurina, Valeriya Ushakova

**Affiliations:** V. Serbsky National Medical Research Centre of Psychiatry and Narcology, 119034 Moscow, Russia; abramova1128@gmail.com (O.A.); zubkov.e@serbsky.ru (E.Z.); olga672@yandex.ru (O.G.); ushakovavm@yandex.ru (V.U.)

**Keywords:** theranostic, Alzheimer’s disease, nanoparticles

## Abstract

Alzheimer’s disease (AD) remains an incurable neurodegenerative disorder. The concept of theranostics—combining diagnostic and therapeutic functions within a single nanoplatform—has been explored for over a decade. Despite a growing number of publications, no theranostic system has yet reached clinical application for AD. This critical review analyzes the fundamental conceptual contradictions that hinder the clinical translation of theranostic nanoplatforms for AD and identifies alternative strategies where nanotechnology may still be beneficial. The review presents key aspects essential for understanding theranostics challenges: AD molecular targets, analysis of existing nanoplatforms, identification of three inherent conceptual conflicts, and viable alternative approaches. Our analysis reveals three core conceptual conflicts: the pharmacokinetic conflict, where diagnostics demand rapid accumulation and clearance while therapy requires prolonged retention—exacerbated by minimal brain delivery (1–2% ID/g) and peripheral toxicity risks; the dose conflict, characterized by orders-of-magnitude disparities between diagnostic and therapeutic dosing, rarely quantified for identical particles; and the temporal conflict, pitting one-time diagnostics against chronic therapy needs, as long-persisting particles generate irremovable brain background signals. We further identify a pervasive methodological trap: predominant focus on mature β-amyloid (Aβ) fibrils overlooks soluble oligomers as the primary toxic species. We conclude by proposing viable alternatives: preclinical intervention for time-limited “hit-and-clear” applications; coordinated theranostic monitoring with separate diagnostics/therapy; theranostic pairs using ligand-matched, function-optimized particles; and external stimuli for temporal function separation. A practical roadmap guides the transition from conceptual demonstrations to clinical translation. Addressing these contradictions can transform theranostics from elegant chemical constructs into clinically meaningful AD tools.

## 1. Introduction

The growing proportion of the elderly population and the rising prevalence of dementia represent major challenges in modern healthcare. According to World Health Organization data, approximately 57 million people worldwide lived with dementia in 2021, with nearly 10 million new cases each year [1]. Alzheimer’s disease (AD) is the most common type of dementia, accounting for 60–80% of cases [2]. The disease not only significantly reduces the quality of life of patients and their families but also imposes a substantial economic strain [3].

AD pathogenesis involves multiple interconnected mechanisms. Hallmarks include extracellular senile plaques composed of β-amyloid (Aβ) and intracellular neurofibrillary tangles of hyperphosphorylated tau protein [4]. Dysregulated processing of amyloid precursor protein (APP) generates toxic Aβ species, triggering oxidative stress, mitochondrial dysfunction, calcium dysregulation, and neuroinflammation [5]. Tau hyperphosphorylation disrupts microtubule stability, promotes intraneuronal aggregates, synaptic dysfunction, and neuronal loss [5,6]. Chronic neuroinflammation—driven by activated microglia and oxidative stress from protein aggregates—creates a vicious cycle that worsens proteopathy and impairs autophagy [7].

AD diagnosis relies on detecting amyloid and tau pathology via positron emission tomography (PET), magnetic resonance imaging (MRI), and cerebrospinal fluid (CSF) analysis [8,9,10]. However, PET imaging of amyloid plaques has low specificity [11], MRI cannot detect early changes, and CSF analysis is invasive. Moreover, pathological changes in the brain begin to accumulate many years before the appearance of clinical symptoms [12], highlighting the problem of early non-invasive diagnosis.

Current AD therapy is limited to symptomatic treatment [13]. Cholinesterase inhibitors (e.g., donepezil, rivastigmine) and memantine modestly support cognition but do not target core pathology [14]. Recent advances include Food and Drug Administration (FDA)-approved anti-Aβ monoclonal antibodies (lecanemab, donanemab), which clear amyloid but yield limited slowing of cognitive decline and carry risks such as amyloid-related imaging abnormalities [15,16,17].

Methods that combine the capabilities of early specific diagnosis and therapeutic intervention are extremely relevant, as they make it possible to detect pathology at early stages with a moderate degree of neural tissue degradation and initiating treatment in a timely manner. Theranostics—one such strategy—has gained traction over the past two decades.

The term “theranostics” was introduced into scientific use in 2002 by John Funkhouser to describe agents that can be used simultaneously for diagnosis and therapy [18]. In the modern concept, the theranostic approach is most often implemented using nanoscale carriers that make it possible to simultaneously identify the pathological focus and deliver a therapeutic agent to it. Core components include a carrier (e.g., nanoparticle for transport/protection), a diagnostic agent (e.g., fluorescent dye or radioisotope for visualization), and therapeutic agent [19]. The use of such a diagnostic–therapeutic platform allows for real-time tracking of pharmacokinetics and biodistribution, as well as local action directly in the area of the pathological focus [20].

Various types of nanoparticles (Nps) are used in research—both organic (lipid-based, polymeric) and inorganic (magnetic, gold, carbon-based) [20,21,22]. Each class possesses unique properties: lipid particles (liposomes, exosomes) offer biocompatibility [23,24,25]; polymeric particles (Poly(lactic-co-glycolic acid) (PLGA), dendrimers) provide biodegradability and functionalization [19,26]; superparamagnetic iron oxide Nps (SPIONs) enable MRI contrast and magnetic targeting [27]; and gold and carbon-based particles, including quantum dots (QDs) support photothermal therapy and protein aggregate disruption [21,28,29,30]. The high interest in these approaches is confirmed by the exponential growth in the number of publications over the past 10–15 years [21,31].

To date, theranostics has been most successfully implemented in oncology [32]. A successful example is the radiopharmaceutical ^177^Lu-DOTATAT, which simultaneously performs diagnostic and therapeutic functions in the treatment of neuroendocrine tumors [33]. The key success factor is the presence of solid tumors with disrupted vascular architecture, which provides a pronounced enhanced permeability and retention (EPR) effect [32,34]. Due to strong vascularization and increased vascular permeability in the tumor area, theranostic systems can successfully accumulate in the target region, exerting a long-term therapeutic effect. Related to these properties, several theranostic drugs have already been approved by the FDA for cancer therapy [35].

Nevertheless, despite the multimodality and promise of theranostic approaches, theranostics face underemphasized limitations in neurodegeneration, distinct from oncology. AD features diffuse pathology, blood–brain barrier (BBB) restrictions, and hypovascularized lesions that hinder delivery. Furthermore, in neurodegeneration, patients require lifelong therapy, which poses major challenges regarding appropriate dosing, toxicity assessment, elimination of nanodrugs, and the uniformity of the diagnostic and therapeutic approach. The risk of chronic toxicity and accumulation of non-degradable particles in the brain significantly increases for patients with AD [36]. This calls into question the universality of theranostic technology and requires deep adaptation of strategies considering the realities of chronic neurodegenerative disease.

Thus, despite theranostic agents being a rapidly developing field, no theranostic system has yet entered clinical evaluation for AD. While nanoparticle-based drug delivery systems for AD have reached clinical trials—for example, APH-1105 intranasal Nps, which completed Phase 2 as an alpha-secretase modulator—these platforms lack a diagnostic imaging component [37]. Most reviews emphasize chemistry over biology and overlook AD-specific conceptual barriers.

Therefore, the aim of this review was a critical analysis of the conceptual reasons hindering the clinical translation of theranostic Nps for AD, and the search for alternative strategies for their application in diagnosis and therapy using nanotechnology.

## 2. Molecular Targets of Theranostics

**Figure 1:**
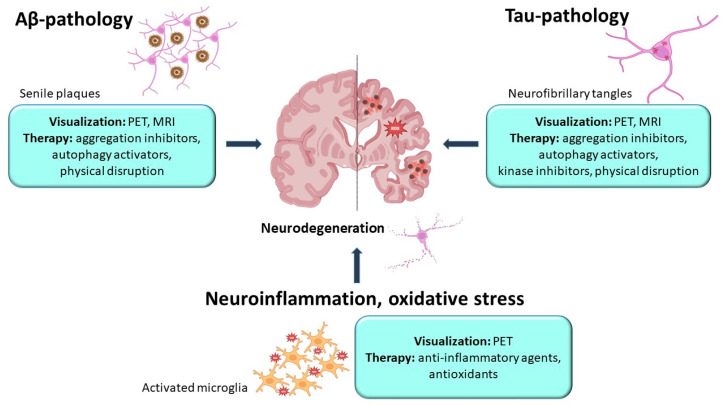
Molecular targets for theranostic intervention in AD for future clinical application. PET—positron emission tomography, MRI—magnetic resonance imaging. Created in Biorender. Zorkina, Y. (2026) https://BioRender.com/ry8uv36 (accessed on 27 March 2026).

A pivotal challenge in developing effective theranostic platforms is selecting pathogenetically relevant molecular targets that are accessible for imaging and amenable to therapeutic modulation. In AD, primary targets for theranostic nanodrugs encompass amyloid pathology, tauopathy, and neuroinflammatory processes (Figure 1).

### 2.1. β-Amyloid (Aβ)

The amyloid hypothesis has historically been assigned a key role in the pathogenesis of AD [38]. Aβ aggregation into oligomers, fibrils, and senile plaques initiates a neurotoxic cascade, including tau hyperphosphorylation, mitochondrial dysfunction, and neuroinflammation [5]. Numerous preclinical studies target amyloid with theranostic nanoplatforms [39,40]. The accumulation of Aβ in the brain can be visualized in vivo using PET with FDA-approved radiopharmaceuticals [27,41]; approaches aimed at MRI diagnostics using magnetic Nps are also actively used [42]. Targeting employs Aβ-specific ligands (e.g., anti-amyloid antibodies, curcumin, Congo red, thioflavin) [43]. Therapeutic effects arise from aggregation inhibitors (e.g., methylene blue), physical disruption methods, and agents promoting autophagy or Aβ clearance [44,45].

Despite decades of research and comparatively successful results from preclinical trials, the effectiveness of therapy targeting Aβ remains contentious. Clinical trials often fail to deliver benefits. For example, bapineuzumab and solanezumab showed no cognitive improvement in Phase 3 trials [46,47], while crenezumab also failed to meet primary endpoints [13]. Even recently approved antibodies lecanemab and donanemab yield only modest cognitive slowing and carry significant risks of amyloid-related imaging abnormalities [15,16,17]. Consequently, Aβ’s primacy in pathogenesis is increasingly questioned [48]. Evidence indicates the accumulation of Aβ 10–20 years before the development of a full-blown clinical picture of the disease, which makes late intervention ineffective, and also demonstrates the detection of senile plaques in cognitively preserved patients [49]. This implies either flawed hypothesis or treatment refractoriness over time. Thus, non-amyloid targets—such as tau, inflammation, and oxidative stress—warrant priority [7].

### 2.2. Hyperphosphorylated Tau-Protein

Unlike amyloid-β, tau protein has long remained on the periphery of interest for developers of theranostic systems. However, as noted in their review by Kim et al. [50], targeting tau pathology using Nps represents a promising strategy capable of restoring neuronal transmission and synaptic integrity. Hyperphosphorylated tau accumulation correlates more robustly with cognitive decline and neuronal loss than Aβ [49,51], making it a promising agent for targeted therapy delivery. Visualization employs PET tracers [52,53] and MRI contrast agents [54], though studies lag behind Aβ imaging. For targeted delivery, specific antibodies or vectors are also used, and the therapeutic effect is achieved by suppressing aggregation processes and enzymes that mediate hyperphosphorylation, stimulating autophagy, and using physical methods such as photodynamic dissociation of aggregates [21,55] (Figure 1). Nevertheless, the development of such Nps is currently in its early stages and requires additional research for a thorough assessment of their safety, efficacy, and long-term consequences. One difficulty is the intracellular localization of tau, which reduces the accessibility of its epitopes for nanoprobes. Consequently, the task of optimizing nanoparticle design, distribution methods, and targeting accuracy continues to present significant challenges.

### 2.3. Other Pathogenetic Mechanisms

Alongside protein aggregation, mitochondrial dysfunction—with reactive oxygen species (ROS) accumulation and neuroinflammation driving microglial activation—plays a central role in AD pathogenesis [56]. The translocator protein, a hallmark of activated microglia, serves as the gold standard for PET-based neuroinflammation imaging [57]. The therapeutic potential here can be realized using anti-inflammatory therapy, aimed, for example, at suppressing the NOD-like receptor family pyrin domain containing 3 (NLRP3) inflammasome [26,58]. Of particular interest is the use of Nps with antioxidant activity [59] (Figure 1), due to the important role of oxidative stress in the pathological changes in AD. Nevertheless, despite the promise of targeting neuroinflammation and ROS, it remains unclear whether microglial activation is a primary triggering mechanism or merely a consequence of protein aggregate accumulation [60]. Moreover, anti-inflammatory therapy is generally adjuvant in nature and aims to slow the progression of the disease, rather than eliminate its root cause. This defines the role of Nps with antioxidant and anti-inflammatory properties as an important, but rather adjuvant, component in combined theranostic strategies.

Cholinergic dysfunction, underpinning AD cognitive deficits, offers another target. PET imaging of cholinergic neurons paired with targeted acetylcholinesterase inhibitor delivery shows promise [61,62]. Yet, this deficit is consequential rather than causal within the neurodegenerative cascade. Inhibitors yield symptomatic relief but spare core pathologies like Aβ/tau accumulation and neuroinflammation [14]. Thus, cholinergic targeting suits multimodal platforms as an adjunct, not standalone theranostic focus.

Another potential target for theranostic intervention is the neurodegenerative process itself—the progressive loss of neurons. For instance, Zhang and colleagues developed traceable polymeric Nps designed to modulate neural stem cells (NSCs) and promote their differentiation into neurons, with SPIONs enabling MRI monitoring of brain accumulation. This system improved cognitive performance by steering NSC differentiation toward a neuronal phenotype [63]. While this regenerative approach differs from anti-aggregation or anti-inflammatory strategies, it represents a conceptually distinct pathogenetic entry point—aiming to replace lost neurons rather than halt protein aggregation.

From this perspective, strategies for multimodal systems that can combine several diagnostic or therapeutic approaches deserve special attention. An example is the development of a multifunctional nanocomposite based on mesoporous silica, containing a targeting ligand for phosphorylated tau, magnetic crystals for MRI visualization, methylene blue to suppress protein aggregation, and an antioxidant component [64]. This approach allows targeting several key links of the pathological cascade at once: from primary protein aggregates to secondary processes of inflammation and oxidative stress originating from mitochondrial dysfunction.

Given AD’s heterogeneous, interconnected pathologies, multimodal theranostic platforms—targeting multiple nodes—represent not merely a promising avenue but an essential evolution beyond monotherapies.

### 2.4. Methodological Trap: Fibrils vs. Oligomers

Existing methods of AD therapy, including acetylcholinesterase inhibitors and NMDA receptor antagonists, are symptomatic in nature and do not affect key links in the pathogenesis. In this context, the development of Nps inhibiting amyloid-β aggregation appears promising [19]. However, a critical examination of the methodology used in such studies reveals a conceptual gap: a profound mismatch between the molecular target being pursued (mature fibrils) and the species that actually drives neurotoxicity (soluble oligomers).

The most toxic forms of Aβ are not mature fibrils, but soluble oligomers [65]. Structural studies highlight their differences: heterogeneous, disordered oligomers contrast with ordered fibrils [66]. Thus, the KW1 antibody, which selectively binds Aβ(1–40) oligomers, recognizes a hydrophobic epitope in the region of residues 18–20, while the fibril-specific antibody B10 interacts with charged patterns on the fibril surface [66]. This means that methods tuned for fibril detection cannot be automatically transferred to oligomers. Moreover, KW1 even distinguishes different types of oligomers—for example, those formed by Aβ(1–40) and Aβ(1–42)—which emphasizes the need for careful validation of selectivity when developing theranostic agents.

Despite these fundamental differences, the vast majority of studies continue to rely on methods oriented towards fibrils. This persistent strategy can be explained by several factors. First, fibrils are historically the most visible and well-characterized AD pathology, and the amyloid cascade hypothesis originally centered on plaque deposition. Second, fibrils are easier to detect experimentally using routine methods such as thioflavin T (ThT) and PET tracers, which are widely available and standardized. Third, oligomers are heterogeneous, transient, and structurally less defined, making them more challenging to target and quantify. Thus, the predominance of fibril-directed research reflects methodological convenience and historical inertia rather than a deliberate choice based on pathogenic relevance.

The main tool remains ThT—a dye that specifically binds to the β-sheet structure of fibrils. At the same time, an important observation is that the ThT signal can be registered even in the absence of mature fibrils: in the presence of the oligomer-specific antibody KW1, only non-fibrillar aggregates are formed, yet the ThT signal continues to increase [66]. Thus, ThT is not a strictly specific marker of fibrils, and its use as the sole criterion for therapy efficacy is incorrect.

The limitations of fibril-oriented approaches are also confirmed at the clinical level. A Cochrane systematic review by Martinez and co-authors [11], which assessed the diagnostic accuracy of PET with 18F-florbetapir (a ligand for fibrillar amyloid) for predicting the progression of mild cognitive impairment to dementia in AD, demonstrated that with good sensitivity (89%), the specificity of the method is only 58%. This means a high level of false-positive results (36 per 100 examined), which makes the test unsuitable for routine clinical use. This result confirms that focusing on the final, easily detectable forms of Aβ aggregation (fibrils) fundamentally limits both the diagnostic and prognostic value of the method.

In response to this problem, attempts have been made in recent years to directly measure the interaction of Nps with oligomers. Thus, Liu and co-authors [67] provide quantitative dissociation constants for oligomers (68.3 nM) and fibrils (112.5 nM) of Aβ, which allows for an objective assessment of the affinity and selectivity of the developed platform. Using classical mesoporous silicon Nps (50–100 nm) with a magnetic core, the authors implemented a full-fledged theranostic system: MRI visualization due to Fe_3_O_4_, targeted delivery due to transferrin, and therapeutic action due to the binding and elimination of Aβ. However, even in this work, despite the correct measurement of constants, the question remains open as to how exactly binding to oligomers affects their toxicity and subsequent fate in the brain.

Another example of a quantitative approach is presented in the work of Wang and co-authors [68]. The authors measured the dissociation constants for chitosan–hyaluronic acid Nps (CHG NPs) and showed that the affinity for oligomers (Kd = 5.0 μg/mL) is slightly higher than for fibrils (Kd = 6.2 μg/mL). Although this difference is small and does not indicate high selectivity, the kinetic confirmation that the particles do interact with oligomers is fundamentally important. Using kinetic experiments, it was demonstrated that CHG NPs detect oligomers already after 3 h of incubation, while the ThT signal, reflecting fibril formation, only begins to increase after 14.6 h. As the authors themselves note, the increase in CHG NP fluorescence at early stages of incubation is associated with binding to soluble oligomers. This serves as direct confirmation that ThT does not see oligomers—the most toxic forms of Aβ. However, even in this work, despite the correct measurement of binding to oligomers, the dose conflict detailed in Section 4.2 persists.

These findings are not actually contradictory [67,68]. The explanation lies in the fact that some non-fibrillar aggregates—including certain oligomeric conformations—can acquire β-sheet-rich regions that are still recognized by ThT, even in the absence of mature fibrils. Thus, a positive ThT signal does not necessarily prove the presence of mature fibrils, but it also does not prove selective binding to toxic oligomers. ThT is therefore not a strictly specific marker of fibrils, and using it as the sole criterion for therapeutic efficacy is methodologically incorrect.

Another example of a system targeting oligomers are W20/XD4-SPIONs, where the oligomer-specific antibody W20 does not bind monomers and fibrils, and the XD4 peptide additionally activates microglia for phagocytosis of oligomers [63]. This is one of the few examples where selectivity for oligomers is built into the design from the very beginning.

Without direct data on the interaction of Nps with oligomers, it is impossible to assess their theranostic potential. A system that detects only fibrils may miss the earliest stages of pathology, reducing diagnostic value. Therefore, standardization of the methodology for evaluating theranostic systems is necessary, including mandatory verification of the effect on oligomeric forms of Aβ using specific detection methods (A11—recognizing the common conformation of oligomers of different amyloidogenic proteins, dot-blot, FRET) and toxicity analysis on neuronal cultures. The emergence of cryo-EM structures of Aβ opens up the possibility for computer-aided design of more selective theranostic agents [65].

## 3. Analysis of Existing Nanotheranostic Systems for AD

### 3.1. General Characteristics and Classification of Theranostic Systems

For this review, we conducted a systematic literature search in three electronic databases: PubMed, Scopus, and Web of Science, covering the period from January 2015 to December 2024. The search strategy combined terms related to AD, nanomedicine, and theranostics using Boolean operators. The following keyword groups were applied to titles, abstracts, and keywords: (“Alzheimer disease” OR “AD” OR “amyloid beta” OR “tau”) AND (“theranostic” OR “theragnostic” OR “diagnostic and therapeutic”) AND (“nanoparticle” OR “nanoplatform”).

Inclusion criteria were: original research articles published in peer-reviewed journals; studies explicitly demonstrating dual functionality of the same nanoparticle platform—diagnostic and therapeutic effects; studies focused on AD (in vitro or in vivo models).

Exclusion criteria were: studies addressing only diagnosis or only therapy without the other function (e.g., imaging-only or therapy-only Nps); reviews, conference abstracts, editorials, or book chapters; studies on other neurodegenerative diseases without AD relevance.

Given the conceptual rather than exhaustive nature of this critical review, we selected representative examples that best illustrate the identified contradictions and the diversity of nanoparticle platforms (polymeric, lipid, carbon dots, magnetic, gold). The final selection of 23 studies is presented in Table 1.

The leading position in the field of AD nanotheranostics is occupied by polymer Nps and polymer-based nanocomposites. The popularity of this platform is due to the high flexibility of chemical design: the polymer matrix allows for the encapsulation of various therapeutic agents (e.g., methylene blue [64], cyclophosphamide [70], curcumin [71]), and the surface can be easily functionalized with targeting ligands and contrast agents for MRI or Single-Photon Emission Computed Tomography (SPECT). This design ensures a synergistic therapeutic effect, as demonstrated in the work of Chen et al. [64], where the combination of a tau aggregation inhibitor and an antioxidant led to a significant improvement in cognitive functions in rats.

Carbon dots (CDs) represent another widely employed approach, distinguished by their unique multifunctional properties. Their advantage lies in a unique combination of properties: they possess their own stable fluorescence, which eliminates the need for additional diagnostic labels [68,84]. Their small size (less than 5 nm) and surface rich in functional groups allow them to interact effectively with Aβ-peptides, inhibiting their fibrillation and even disaggregating mature fibrils [77,79]. Furthermore, many CDs exhibit antioxidant activity [78,79,80], positioning them an “all-in-one” platform for combination therapy.

Magnetic Nps (SPIONs and USPIONs) represent another key class of theranostic Nps. Primarily utilized as MRI diagnostic platforms, their functionalizable surfaces enable hybrid systems incorporating therapeutic agents (e.g., rutin [83]) and targeting vectors. A prominent trend involves SPION conjugation with specific antibodies (e.g., the oligomer-specific scFv W20) for early diagnosis and simultaneous activation of phagocytosis by microglia [63].

Liposomes [74,75] and gold nanorods [29,81] exemplify additional theranostic approaches. Liposomes offer biocompatibility and multi-ligand capacity for BBB penetration [74]. Gold nanorods enable near-infrared photothermal therapy to disrupt Aβ aggregates post detection.

Also included in the selection were singular but promising developments, such as self-fluorescent tryptophan Nps [85], pyrimidine-based PET radioligands [86], and a charged dye molecule [84]. These platforms offer distinct advantages that address some of the conceptual contradictions identified in this review. Tryptophan Nps [85] combine intrinsic fluorescence, eliminating the need for external diagnostic labels, with inherent anti-amyloid activity, achieving both Aβ aggregation inhibition and fibril disaggregation. Their self-fluorescent nature simplifies theranostic design and avoids potential toxicity from additional fluorophores. Pyrimidine-based PET radioligands [86] target the σ1 receptor—a promising target involved in neuroprotection and neuroinflammation. The authors validated these agents in both transgenic mice and non-human primates, demonstrating cross-species translatability. The charged dye molecule DBA-SLOH [84] exhibits high affinity for Aβ oligomers, is BBB-permeable without active targeting ligands, and its fluorescence is activated upon binding to Aβ. Its dual functionality as both a diagnostic probe and an aggregation inhibitor positions it as a minimalist theranostic instrument. Together, these examples illustrate that non-canonical platforms—moving away from complex multicomponent Nps—may offer unexpected solutions to the pharmacokinetic and dose conflicts discussed below.

Of particular interest in the field of nanotheranostics are hybrid Nps, which combine components of different chemical natures in a single platform [64,69]. For example, Chen et al. [64] developed a CeNC/IONC/MSN-T807 nanocomposite integrating superparamagnetic iron oxide nanocrystals (IONCs) for MRI/PET bimodal imaging, nanoceria (CeNCs) as an antioxidant, and methylene blue as a tau aggregation inhibitor, loaded into mesoporous silica Nps. This design achieves true synergy: the magnetic component enables diagnosis, while the cerium and methylene blue provide complementary therapeutic mechanisms (antioxidant + anti-aggregation). Similarly, Cui et al. [69] constructed UCHQs—upconversion luminescence Nps (NaYF_4_:Yb/Er/Tm) coated with DSPE-PEG and functionalized with 8-hydroxyquinoline-2-carboxylic acid (HQC). Here, the upconversion core provides luminescence imaging, while HQC chelates Cu^2+^ ions, simultaneously reducing metal-induced oxidative stress and inhibiting Cu^2+^-catalyzed Aβ aggregation. The hybrid architecture thus enables multimodal imaging and combination therapy within a single nanoplatform, addressing the multifactorial nature of AD pathology more effectively than single-component systems.

The main target for the vast majority of theranostic systems remains Aβ: most of the analyzed studies were directly or indirectly aimed at various forms of Aβ—from monomers and toxic oligomers to mature fibrils and plaques. Researchers employ two primary approaches: detection of Aβ (fluorescence, MRI, PET, SPECT) and modulation of its aggregation (inhibition of fibrillogenesis and/or disaggregation of existing fibrils). Tau protein serves as a target far less frequently, where systems combine hyperphosphorylated tau targeting with therapies reducing oxidative stress and inhibiting Aβ aggregation [64,72]. Oxidative stress and neuroinflammation rank as key secondary targets (10 studies), with Nps incorporating antioxidants (CeNCs, tannic acid, curcumin, quercetin) or immunosuppressants (dexamethasone, cyclophosphamide) to prevent neuronal death and attenuate inflammation [58,70,71,78].

MRI emerges as the preferred in vivo diagnostic modality, owing to its clinical relevance and compatibility with SPIONs or Gd-chelates for visualization. Fluorescence imaging (including bioluminescence and near-infrared) also features, primarily with cell cultures and small animals, due to its sensitivity and simplicity. Validation of the systems’ effectiveness was carried out predominantly on genetic AD models (transgenic mice), as well as on cell cultures (SH-SY5Y, PC12, BV-2) and alternative models (nematode *C. elegans*, rats with induced pathology).

Thus, the selection demonstrates an active search for multifunctional Nps, in which polymer dominate due to their versatility. The main vector of development is aimed at targeting Aβ in combination with the correction of oxidative stress, with a steady trend towards the creation of “two-in-one” systems combining therapy with highly sensitive diagnostics for a personalized approach to the treatment of AD.

### 3.2. Analysis of Functional Characteristics of Theranostic Systems

#### 3.2.1. Diagnostics and Specificity of Detection

In all analyzed studies, a diagnostic component is present, implemented using a wide range of imaging methods. Fluorescence imaging is most frequently used [68,77,79,84], due to the simplicity of implementation and the high sensitivity of the method, allowing the detection of pathological aggregates both in vitro and in small animals. A significant portion of the studies use MRI with contrast agents based on gadolinium [70,71], SPIONs [58,63,73,83], or manganese ions [72], reflecting the researchers’ desire to create platforms potentially applicable in clinical practice. A number of studies employ multimodal approaches, combining MRI with PET [64], SPECT [70,71], or fluorescence imaging [72,75], which compensates for the limitations of each method individually.

The specificity of the diagnostic signal is ensured by various targeting strategies. The most common approach is the conjugation of Nps with antibodies or their fragments specific to various forms of Aβ [63,70,71,81]. Alternative strategies include the use of peptide ligands targeting hyperphosphorylated tau protein [64] or the property of penetrating the BBB [58,72], as well as small molecules that bind to the beta-sheet structure of amyloid fibrils [83]. In some works, specificity is achieved through electrostatic interactions between the charged surface of Nps and negatively charged amyloid aggregates [68]. In most studies, targeting specificity is confirmed by methods of colocalization with dyes, immunocytochemistry, or quantitative assessment of accumulation in target tissues.

The sensitivity of diagnostic methods is quantitatively characterized in only a portion of the studies. Wang et al. [68] reported a limit of detection for beta-amyloid oligomers at the nanomolar level, and Ruan et al. [58] demonstrated the possibility of plaque visualization that more accurately reveals the actual deposition of Aβ than previously used methods. Hu et al. [83] showed a higher contrast-to-noise ratio on MRI after the administration of targeted particles, and Liu et al. [63] demonstrated the possibility of differentiating transgenic animals from healthy controls using MRI.

A direct comparison of the developed methods with existing diagnostic standards is present in only a few studies. Wang et al. [68] showed the advantage of the developed Nps in detecting toxic oligomeric forms of beta-amyloid, compared to thioflavin. Ruan et al. [58] were the first to demonstrate the possibility of three-dimensional quantitative assessment of plaque burden using MRI, which is unavailable with standard microscopy. In general, the diagnostic component in most studies serves to demonstrate the concept of targeted delivery and visualization, rather than to create a competitive diagnostic product.

#### 3.2.2. Therapeutic Strategies and Their Effectiveness

The therapeutic component is implemented through diverse mechanisms of action. The most common approach is the inhibition of Aβ aggregation and the disaggregation of already formed fibrils [29,68,77,79,81,84]. Many studies also include an antioxidant component aimed at reducing the level of ROS [64,72,78,80,83], and an anti-inflammatory effect, implemented through the delivery of immunosuppressants [70,71].

Much less frequently, the target of therapy is tau protein, although the works of Chen et al. [64] and Gu et al. [72] demonstrated a reduction in its hyperphosphorylation due to antioxidant action or activation of neuroprotective signaling cascades. Individual studies implement more complex therapeutic strategies, including the chelation of metal ions [69], photothermal disruption of fibrils under the action of near-infrared radiation [29,81], control of stem cell differentiation [73], or activation of microglial phagocytosis [63].

Current understanding of the pathogenesis of AD points to soluble Aβ oligomers as the main neurotoxic form of the peptide, responsible for disrupting synaptic plasticity and triggering neurodegeneration in the early stages of the disease [68]. Among the analyzed theranostic systems, Nps capable of selectively recognizing oligomers are of particular importance. Wang et al. developed CHG NPs, detecting oligomers with a limit of 0.1 nM, which is unattainable with standard dyes [68]. Liu et al. created the W20/XD4-SPION system with the oligomer-specific antibody W20, which does not bind monomers and fibrils, and the XD4 peptide additionally activates microglia for phagocytosis of oligomers [63]. The DBA-SLOH molecule also exhibits affinity for oligomers (Kd = 2.77 μM) and can effectively bind to them [84]. Thus, the development of systems targeting oligomers opens up opportunities for early diagnosis and therapy acting on the most toxic forms of the peptide.

The effectiveness of therapy is confirmed in most studies on cellular models, where a significant increase in the survival of neuronal cells upon exposure to toxic concentrations of Aβ is shown [29,64,72,76,77,78,80,83]. In animal models, a reduction in the burden of amyloid plaques [58,68,75], a decrease in neuroinflammation and oxidative stress, as well as an improvement in cognitive functions in behavioral tests [58,64,72,73,83,85] have been demonstrated. In nematode models, a significant prolongation of lifespan upon the administration of Nps is shown [68,77,78,79,80,81].

A comparison with monotherapy was conducted in some of the studies and shows the advantage of Nps over free therapeutic agents. Agyare et al. [70] showed that encapsulation of cyclophosphamide in Nps allows for a greater anti-inflammatory effect. Hu et al. [83] proved that targeted delivery and controlled release provide an advantage over non-targeted systems.

#### 3.2.3. Integration of Diagnostics and Therapy

Analysis reveals that true synergy between diagnostic and therapeutic components occurs in few studies. Many represent parallel function combinations within single particles, where diagnostics and therapy operate independently [70,71,74]. Several demonstrate genuine synergy, wherein components interact cooperatively. Compelling examples include systems where pathological target binding simultaneously activates diagnostic signaling and therapeutic action—as in Cui et al. [69], where copper chelation serves dual detection/treatment roles, and Li et al. [29] and Liu et al. [81], where post-binding photoirradiation induces localized heating and fibril disruption.

Another form of synergy is the multicomponent interaction of various therapeutic mechanisms within a single particle. In the works of Chen et al. [64], Gu et al. [72], and Zhang et al. [75], antioxidant, anti-inflammatory, and anti-amyloid effects complement each other, providing a complex impact on the pathogenesis of the disease. Systems with controlled release of the therapeutic agent in response to specific triggers of the pathological microenvironment, such as an increase in the concentration of hydrogen peroxide [83], deserve special attention. In these cases, a diagnostic marker of the pathology becomes the direct activator of therapy.

Upon closer examination, CD-based platforms [77,78,79,80] exemplify parallel function combinations rather than true synergy, as their imaging and therapeutic capabilities operate independently without mutual enhancement. Although the same particle provides fluorescence imaging upon binding to the target and simultaneously inhibits aggregation and neutralizes ROS, these functions do not mechanistically enhance each other. A different example of molecular integration is found in Li et al. [84], where binding of the charged dye DBA-SLOH to Aβ simultaneously triggers fluorescence turn-on and blocks aggregation—yet this also represents parallel functionality rather than true synergy, as the diagnostic signal does not amplify the therapeutic effect.

This analysis demonstrates that most studies establish the conceptual feasibility of multifunctional Nps for AD diagnosis and therapy. Leading examples exhibit true component synergy [64,69,83]. However, experimental designs rarely enable assessment of clinical superiority over separate agent combinations. Absent standardized protocols and comprehensive pharmacokinetic/toxicity data impede preclinical advancement. Among the platforms surveyed, CDs [78,79] and triggerable-release systems [83] appear particularly promising for further development, as they address two key challenges identified in our analysis: CDs combine intrinsic fluorescence with therapeutic activity in a single ultrasmall particle (bypassing the need for additional diagnostic labels), while triggerable-release systems enable on-demand therapy activation using pathological signals (e.g., H_2_O_2_), thereby mitigating the dose and pharmacokinetic conflicts.

Despite the impressive progress in the development of multifunctional Nps, the conducted analysis reveals a number of systemic limitations hindering the clinical translation of the developed systems. These limitations, including fundamental contradictions in pharmacokinetics, dosing, and the temporal organization of therapy, will be discussed in detail in the next section.

## 4. Conceptual Contradictions of Theranostics in AD

**Figure 2:**
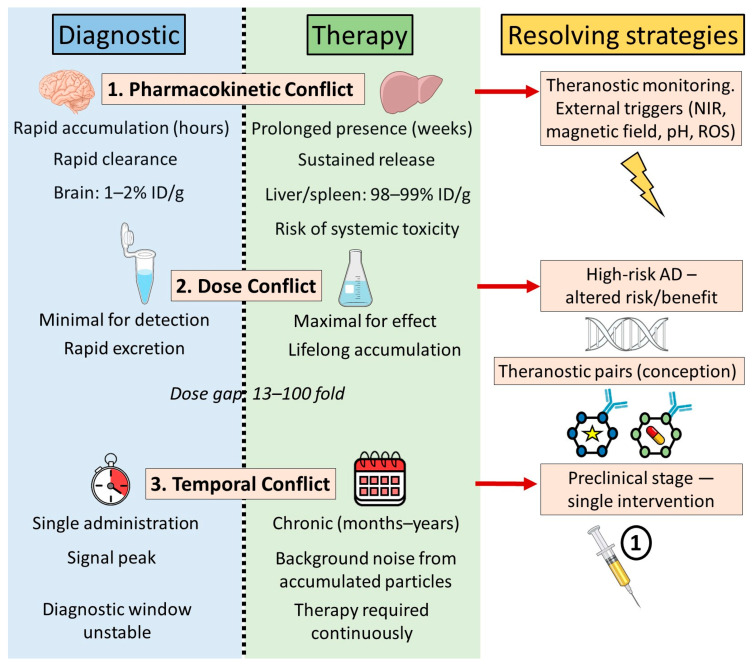
Three inherent conflicts of theranostic platforms in AD and potential strategies to address them. The figure summarizes the fundamental contradictions that arise when attempting to combine diagnostic and therapeutic functions in a single nanoplatform. Pharmacokinetic Conflict: Arises from opposing requirements for rapid accumulation and clearance (diagnostics) versus prolonged presence and sustained release (therapy). This is compounded by the extremely low fraction of the dose reaching the brain (1–2% ID/g), while the vast majority accumulates in peripheral organs (98–99% ID/g), creating a risk of systemic toxicity. Dose Conflict: Reflects the discrepancy between the low doses sufficient for sensitive detection (minimal for detection) and the high doses required for a therapeutic effect (maximal for effect). Temporal Conflict: Highlights the incompatibility between single or short-term diagnostic interventions and the need for chronic, lifelong therapy. A single diagnostic peak can be obscured by background noise from accumulated particles, and the diagnostic window itself is unstable due to the dynamic nature of the BBB over the course of the disease. Resolving strategies are shown below each conflict: External triggers (near infrared (NIR) irradiation, magnetic field, pH, ROS) can separate diagnostic and therapeutic functions in time. High-risk AD subtypes (rapidly progressive/genetic) shift the risk/benefit balance, justifying higher-risk interventions. Preclinical stage offers a window for single, time-limited interventions. Theranostic monitoring involves sequential diagnosis, therapy, and re-assessment. Theranostic pairs (conceptual) propose using two particles with a shared ligand, each optimized for its task. Some icons in this figure were adapted from BioArt (https://bioart.niaid.nih.gov).

Analysis of existing theranostic systems naturally raises a critical question: why do even promising concepts lack data essential for assessing true potential? A systematic check of even a limited selection of original studies reveals consistent methodological gaps: accumulation in the brain is extrapolated from fluorescence without quantitative measurement (%ID/g); diagnostic/therapeutic doses reported in disparate units or for separate components; absent direct comparisons with individually administered agents; and unexamined central nervous system (CNS) elimination. These represent not isolated flaws but field-wide standards confining publications to “conceptual demonstrations.” Further analysis therefore inevitably takes the form of a reconstruction of implicit contradictions—we are forced to piece together data from those few studies where the authors have at least partially gone beyond the framework of a concept demonstration, to show that even in the best cases, the fundamental conflicts remain unresolved. Three inherent conflicts of theranostic platforms in AD are showed in Figure 2.

### 4.1. Pharmacokinetic Conflict

A fundamental challenge in combining diagnostic and therapeutic agents within single Nps lies in their conflicting pharmacokinetic requirements. Diagnostic agents demand rapid target accumulation within hours, minimal effective doses for signal detection, and swift systemic elimination. Therapeutic agents, conversely, require sustained accumulation and prolonged release for extended efficacy. Nps must maintain long-term therapeutic concentrations in the target organ, penetrating and retaining drugs within the brain while minimizing excretion to preserve effectiveness.

In AD, these conflicts intensify due to target-specific demands. Diagnostics necessitate ultrasensitive early detection of Aβ oligomers—pre-symptomatically—requiring rapid plaque access, clear signals (fluorescence, MRI contrast, PET), and swift clearance to avoid confounding therapy or monitoring. Therapy, conversely, demands prolonged brain retention of active agents to continuously suppress pathology: inhibiting aggregation, disassembling fibrils, or shielding neurons from amyloid toxicity. Nanocarriers must enable weeks-long slow release to sustain therapeutic concentrations, not hours.

Theranostic platform development thus requires balancing these opposing pharmacokinetic demands. As Moorthy et al. [19] note, resolution strategies include: unifying functions within single molecules, external control for mode switching, or programmable multi-stage action temporally separating diagnosis from therapy. Each approach offers validated examples demonstrating how fundamental contradictions can be overcome through nanoparticle design.

The first approach—combining functions in a single agent—is implemented, in particular, in systems based on graphene QDs conjugated with tramiprosate. The QD themselves possess stable fluorescence, which allows them to be used for imaging amyloid aggregates. Tramiprosate, in turn, inhibits Aβ aggregation, acting on different parts of the pathological cascade. Diagnostics and therapy work in parallel here and do not interfere with each other, since they are implemented by different molecular mechanisms within a single particle. The pharmacokinetics of such a system is determined by the nanocarrier, which ensures the delivery of both components across the BBB and their joint accumulation in the target area [87].

A comparable example involves curcumin Nps (FCur NPs). Curcumin offers dual anti-amyloid/anti-inflammatory therapeutic potential alongside Aβ-binding fluorescence. However, its weak native fluorescence, poor bioavailability, and BBB impermeability limit utility. Encapsulation in ultrasmall (~11 nm) Pluronic F127 Nps increased brain accumulation 6.5-fold versus free curcumin while preserving amyloid plaque binding [40]. The pharmacokinetic conflict resolves through nanocarrier-mediated delivery/bioavailability enhancement, with target-activated diagnostic signaling from curcumin itself. Nevertheless, the weak fluorescence intensity may limit the use of such particles for in vivo imaging, which requires additional functionalization with diagnostic labels (e.g., for MRI or PET) [40].

The second approach—external stimulus-triggered mode switching—employs graphene oxide functionalized with thioflavin-S (ThS). ThS specifically binds amyloid fibrils and fluoresces, providing continuous diagnostic signaling. Therapeutic action activates via near-infrared (NIR) irradiation: graphene oxide absorbs NIR light, generating localized heat to disassemble fibrils. This strategy’s key advantage lies in on-demand therapy activation without sustained high therapeutic concentrations. Continuous diagnostics enable real-time efficacy monitoring while minimizing systemic toxicity [30].

The most complex example of resolving the pharmacokinetic conflict is the nanosweeper concept, implemented on the basis of the chitosan polymer. Such a nanoparticle contains two functional elements: the KLVFF peptide, which ensures the capture of Aβ, and the Beclin-1 protein, which activates autophagy. The particle also incorporates a fluorophore for visualization. Its mechanism features programmed two-stage action: initial amyloid plaque accumulation (diagnostic phase, confirmed via fluorescence), followed by cellular internalization and autophagy activation—enabling digestion of both amyloid and the expended particle. This achieved 40–60% Aβ reduction in mouse brains (soluble Aβ: 585 to 190 ng/mg; insoluble: 1539 to 914 ng/mg) [44]. Diagnostics and therapy thus temporally separate: accumulation/visualization precedes therapeutic action, with particle pharmacokinetics optimized for this biphasic behavior—reconciling rapid diagnostic clearance with requisite therapeutic persistence [44].

Thus, despite the fundamental contradiction in the pharmacokinetic requirements for diagnostic and therapeutic agents, modern nanotechnology offers several strategies to overcome it. The choice of a specific approach depends on the nature of the target, the available external stimuli, and the desired ratio between diagnostic sensitivity and the duration of the therapeutic effect. All successful examples share a common trait: theranostic platforms transcend mere summation of diagnostic and therapeutic modules, evolving into integrated systems where developers systematically resolve complex pharmacokinetic conflicts.

A key element of the pharmacokinetic conflict, requiring separate consideration, is overcoming BBB. Delivery strategies can be divided into three main approaches, taking into account the pathological changes to the BBB in AD [88]. The first is the use of receptor-mediated transport (RMT), for example, via the transferrin receptor or LRP1 (angiopep-2 peptide). The second is passive accumulation through areas with disrupted tight junction integrity (analogous to the EPR effect), where the optimal particle size for AD is less than 20 nm. The third approach is neuroinflammation-mediated transport exploiting leukocytes as “Trojan horses” that traverse the BBB to inflammatory sites carrying Nps.

Additional functionalization strategies are discussed in detail in the review by La Barbera et al. [89] and are aimed at active transport strategies (conjugation with ligands for endothelial receptors, e.g., to the transferrin receptor or LRP1) and physicochemical modulation (creating a positive charge for adsorptive-mediated transport, PEGylation to increase circulation time). However, despite the variety of approaches, the accumulation of Nps in the brain remains extremely low. Only a few studies provide quantitative data: for the nanosweeper it was 1.94% ID/g [44], and for mesoporous Nps with dual targeting—1.12–1.57% ID/g [67]. The remaining part of the particles accumulates in the liver, spleen, and lungs, creating a potential toxic burden [20] and distorting the “signal-to-noise” ratio in diagnostic imaging. This makes the quantitative assessment of delivery efficacy a mandatory element of any theranostic study—and at the same time a weak point of the vast majority of works, where accumulation in the brain is either not measured at all or extrapolated from fluorescence data without considering real biodistribution.

### 4.2. Dose Conflict

The next fundamental conceptual contradiction is the conflict of doses used for diagnostics and therapy. For diagnostics, the dose must be minimally sufficient for signal visualization, while for therapy, the dose of the administered drug must be large enough to achieve a therapeutic effect. The dose conflict is indirectly indicated by the extremely small number of publications where the doses of the same nanopreparation for therapy and diagnostics are described. The vast majority of articles on theranostics are conceptual descriptions of a particle capable of both imaging and therapy. Alternatively, authors indicate only one of the doses, usually the diagnostic one. Furthermore, all existing methods of AD treatment are used lifelong, since the disease has a chronic progressive course with a preclinical phase starting 10–20 years before the onset of symptoms, and an inevitable fatal outcome within 5–12 years after their onset [90].

#### 4.2.1. Diagnostic and Therapeutic Doses: Mismatch of Ranges

Even in systematic reviews that specifically select literature on AD theranostics, it is not possible to find examples with complete dose data for a single system.

An analysis of the systematic reviews by Villalva and co-authors [91] on QD and Aminyavari and co-authors [92] on SPIONs showed that neither provides an example of a nanoparticle for which both diagnostic and therapeutic doses were quantitatively determined within a single study. In the tables summarizing diagnostic and therapeutic approaches, different sets of particles appear [91,92]. This means that even when a particle is claimed to be theranostic, researchers typically limit themselves to demonstrating either its imaging ability (indicating the corresponding dose) or its therapeutic efficacy, but do not conduct a systematic quantitative comparison of the doses necessary to implement both functions.

Characteristically, the ranges of diagnostic and therapeutic doses given in these reviews practically do not overlap. For QD, diagnostic concentrations range from 50 nM to 15 mg/kg, while therapeutic ones vary from 5 μM to 200 μg/kg [91]. For SPIONs, diagnostic doses in vivo reach 5860 mg/kg, and therapeutic ones—25–100 mg/kg [92]. Moreover, no cited study employed identical particles for both functions in vivo with unified dosing—diagnostic and therapeutic doses are reported for distinct particles in disparate units, precluding direct comparison. This suggests developers adopt suboptimal compromise doses or forgo dosage standardization entirely. The >400-fold diagnostic dose variability further raises safety concerns for imaging-requiring high doses.

A rare exception is the already mentioned work by Luo and co-authors [44], in which concentrations for both diagnostics and therapy are given for the nanosweeper theranostic system. For in vitro imaging, 20 μg/mL of Nps labeled with the Cy5 fluorophore were used, which made it possible to track Aβ capture and complex internalization in cells. For in vitro therapy, the same concentration (20 μg/mL) increased the viability of N2a neuronal cells from 60% to 92.5% under toxic Aβ load. However, for in vivo experiments, the authors were forced to increase the concentration to 200 μg/mL, administered every two days for a month. Moreover, only 1.94% of the administered dose reached the brain parenchyma, and the therapeutic effect was assessed by the reduction in Aβ levels (soluble Aβ decreased from 585 to 190 ng/mg, insoluble—from 1539 to 914 ng/mg). The diagnostic function in vivo remained qualitative: the authors confirmed the presence of particles in the brain but did not determine whether the diagnostic dose (20 μg/mL) was sufficient for imaging in animals. This example clearly demonstrates the dose conflict: in vitro doses for diagnostics and therapy coincide, but in vivo, a tenfold increase in dose and repeated administration are required to achieve a therapeutic effect. At the same time, the fate of 98% of the administered material accumulated in peripheral organs remains beyond discussion.

An example clearly demonstrating the dose conflict are CHG NPs developed by Wang and co-authors [68]. The authors showed that for the detection of Aβ oligomers and fibrils in vitro, the optimal concentration is 7 μg/mL, with a detection limit reaching 0.1 nM. For imaging Aβ plaques in vivo in the *C. elegans* model, 30 μg/mL is sufficient. However, for inhibiting Aβ aggregation, concentrations of at least 90 μg/mL are required, and the maximum effect is achieved only at 720 μg/mL. Thus, the therapeutic dose exceeds the diagnostic one by 13–100 times. The authors honestly record this contradiction but do not propose a solution—which makes their work not so much an example of successful theranostics, but rather an ideal illustration of its fundamental problem. Moreover, the authors note that at high concentrations (360 μg/mL and above), the background signal of the Nps becomes so intense that it hinders sensitive diagnostics. This contradiction has no resolution within this system: the same particle cannot simultaneously provide high detection sensitivity and sufficient therapeutic efficacy. Despite the good methodological level, the experiments were conducted on *C. elegans*, which do not have BBB, so the issue of penetration into the mammalian brain remains unresolved.

A similar gap is demonstrated by other works. For example, in the study by Wei and co-authors [79], R-CD-75 CDs inhibited Aβ aggregation by 79% at a concentration of 2 μg/mL, but 50 μg/mL was required to achieve 99% inhibition, creating a 25-fold gap between the minimally effective and maximally effective dose. Moreover, the authors do not indicate which of these concentrations could be used for diagnostics and which for therapy in vivo.

The only study where two identical doses were administered for therapy and diagnostics in vivo is the work by Ruan and co-authors [58]. SDP@Cur-CRT/QSH Nps at a dose of 5 mg/kg were used both for MRI visualization of amyloid plaques and for therapy (every 4 days for 3 months). Although the single dose coincides, the cumulative therapeutic dose (>100 mg/kg) exceeds the diagnostic one by 20 times. Over 3 months of administration, the authors did not detect any changes in body weight or organ histology. However, histological analysis was performed immediately after the completion of injections, which does not allow judgment on long-term consequences.

#### 4.2.2. The Price of Repeated Administration: Cumulative Toxicity

The next problem related to doses is the potential toxicity of Nps upon repeated administration, necessary for the chronic course of AD. The paradox lies in the fact that the small size of particles, which provides their theranostic potential (e.g., high neuronal uptake of small SPIONs), simultaneously increases the risk of cellular toxicity, since metals, especially iron, play an important role in neurodegenerative diseases [92].

As shown in the review by Kevadiya and co-authors [20], even “neuroprotective” Nps, such as cerium oxide, can cause hepatotoxicity in mice, which confirms the thesis that one cannot endlessly increase the dose “for better therapy” without causing damage to internal organs. Moreover, the authors directly point out the neurotoxicity of a number of Nps: silver Nps are toxic to neurons and astrocytes derived from human embryonic stem cells; titanium oxide disrupts brain development in the fetus; zinc oxide causes necrosis and apoptosis in macrophages. Since 99% of the administered dose accumulates in the liver and kidneys, the risk of systemic toxicity becomes critical. This issue is not raised in most articles. Unlike oncology, where the risk of toxicity is often justified, in AD, which requires long-term treatment, this risk is unacceptable [20].

#### 4.2.3. The Fate of Particles in the Brain: Lifelong Accumulation

A second systematically overlooked issue concerns the fate of Nps post-BBB traversal. Delivery strategies rarely address CNS elimination mechanisms. Non-degradable core particles (gold, iron oxide) binding amyloid plaques face uncertain futures—potentially persisting indefinitely, generating persistent MRI background signals that confound disease progression tracking during serial imaging. Passive accumulation via tight junction disruption proves particularly problematic: particles may trap in perivascular spaces, inaccessible to neurons and non-eliminable. In chronic diseases requiring repeated dosing, this yields escalating background signals. Thus, “successful brain delivery” risks becoming “permanent accumulation sentencing,” undermining long-term monitoring utility. As Aminyavari et al. [92] rightly emphasize, SPION safety demands rigorous pre-clinical determination, with conflicting toxicity data complicating risk assessment.

Characteristically, even in reviews that recognize the importance of elimination and the risks of accumulation during chronic use, these issues are not projected onto the CNS. Thus, Moorthy and Govindaraju [19] emphasize that the size of dendrimers is critical for renal filtration and preventing toxicity associated with accumulation. Authors advocate long-term toxicological studies yet, when addressing brain delivery, merely confirm BBB crossing without examining subsequent brain parenchymal fate. Self-immolative dendrimers offer controlled degradation to mitigate toxicity, but remain disconnected from CNS elimination challenges. This confirms that the problem of “lifelong accumulation” in the brain remains a “blind spot” even in works that declare the importance of pharmacokinetics and safety.

Before the clinical implementation, critical issues of biological safety and efficacy must be carefully considered, assessing not only short-term but also long-term risks to human health. However, this issue is not raised in most articles, creating a gap between the “concept” and “clinical reality.”

### 4.3. Temporal Conflict

The third fundamental contradiction in AD theranostics is temporal, stemming from divergent diagnostic and therapeutic duration requirements. Diagnostics entail one-time or short-term interventions, whereas chronic neurodegenerative therapy demands prolonged, often lifelong administration. This temporal mismatch—largely unaddressed in the literature—poses critical challenges. All approved AD treatments (acetylcholinesterase inhibitors, memantine, monoclonal antibodies) require lifelong use, necessitating repeated theranostic particle dosing (daily, weekly, or monthly). Optimal imaging windows span hours to days; Kevadiya et al. [20] describe systems peaking at 4 days post-administration. This accumulation/clearance window becomes problematic: particles persisting ≥4 days cause signal overlap during monthly monitoring, rendering repeat diagnostics infeasible (accumulation mechanisms detailed in Section 4.2.3).

None of the analyzed studies discuss the possibility of repeated diagnostics using the same particles. If a patient requires annual monitoring of disease progression, does this mean the need for an annual new dose? What will happen to the signal from particles administered a year ago? Since studies on elimination from the CNS are absent, these questions remain unanswered.

Although individual studies include repeated administration over 1–2 months [44,58], the issue of the optimal regimen for chronic therapy is not systematically discussed. The authors do not justify the choice of frequency and duration, do not compare different regimens, and do not assess long-term consequences. Safety monitoring is limited to histology immediately after the experiment, which does not allow judgment on the risks of real lifelong use.

This challenge intensifies due to dynamic BBB permeability. As Han and Jiang [88] review, AD BBB integrity varies by disease stage rather than remaining static. They emphasize that “quantitative characterization of tight junction loss depends on disease progression stage, animal model, and individual differences”. Consequently, the “diagnostic window”—when particles effectively cross the BBB and generate clear signals—proves unstable and unpredictable. Particles optimized for early-stage AD (minimal barrier disruption) may fail in late stages, and vice versa.

The temporal conflict in AD theranostics remains unresolved. Diagnostics require rapid clearance of particles for repeated studies, while chronic therapy requires their long-term presence. This excludes the possibility of annual monitoring without overlapping signals from previous doses. Repeated administrations have been studied only in a few works, without justification of the regimen and assessment of long-term consequences. The problem is compounded by the dynamics of the BBB: its permeability changes as the disease progresses, making any “optimized” particle ineffective at another stage.

## 5. Potential Scenarios for Theranostics in AD

These identified conflicts do not preclude nanotechnology’s role in AD therapy. Rather, they necessitate abandoning the “one particle for everything” paradigm in favor of flexible, coordinated strategies where diagnostics and therapy operate synergistically yet independently within separate carriers [93].

The first—and most promising—scenario involves preclinical-phase intervention. Here, pathological changes (Aβ/tau aggregate accumulation) have commenced, yet irreversible neurodegeneration and cognitive deficits remain absent. Within this therapeutic window, single or time-limited theranostic interventions targeting toxic protein aggregates can not merely slow but delay or prevent clinical manifestation. This paradigm fundamentally alters pharmacokinetic demands: rather than lifelong therapeutic concentration maintenance (triggering dose/temporal conflicts), a single “hit” suffices, enabling subsequent particle clearance without residual diagnostic background signals.

The sole evidence to date validating this approach comes from [67]. Following single intravenous administration of mesoporous Nps (HA-MMSN-1F12) to APP/PS1 mice, authors observed biphasic Aβ42 blood dynamics: initial sharp decline (peripheral pool binding) followed by elevation exceeding baseline by day 9. Correlation analysis confirmed the secondary rise reflected Aβ release from brain plaque depolymerization. This provides direct evidence that single-dose administration can trigger prolonged (≥9 days) CNS amyloid clearance—a fundamental advantage over antibody therapies incapable of accessing intracerebral pools. Precisely for preclinical stages, before maximal amyloid burden, such time-limited interventions offer potential to alter disease trajectory.

The second scenario is associated not with the stage, but with the subtype of the disease. In clinical oncology, therapeutic risk becomes acceptable when the alternative is guaranteed lethality. The same logic applies to rapidly progressive variants of AD (disease duration 3–5 years) and monogenic forms caused by mutations in the *APP*, *PSEN1*, and *PSEN2* genes. For carriers of such mutations, the development of the disease is practically inevitable and proceeds faster than in the sporadic form. Here, the risk/benefit ratio shifts: toxicity, unacceptable in the long-term course of sporadic AD, may be justified in conditions of rapidly progressing pathology, where effective therapeutic alternatives are currently lacking. This alleviates part of the dose conflict, as safety requirements become less stringent.

The third scenario is theranostic monitoring. Modern diagnostics is shifting towards detecting the disease at the preclinical stage using biomarkers [94], which creates a demand for strategies capable not only of treating but also of monitoring therapy effectiveness in dynamics. The answer could be a transition from the “one particle for everything” to a coordinated process in time, where diagnostics and therapy are performed separately but sequentially. A diagnostic nanoparticle is used to select patients in whom the target is present and accessible. Based on the data obtained, a course of therapy is prescribed, and repeated diagnostics allows for an objective assessment of its effectiveness. This model resolves the pharmacokinetic conflict: the diagnostic particle is optimized for rapid accumulation and clearance, the therapeutic one—for long-term action. They do not interfere with each other because they are separated in time.

One method of implementation is the use of external stimuli for on-demand activation of therapy and conducting repeated diagnostics with the same particles. As shown in the review by Moorthy and co-authors [19], such approaches include:

1. Photothermal therapy with an NIR laser, where diagnostics (dye fluorescence) works constantly, and therapy (heating) is activated only after confirmation of the target’s presence (e.g., the graphene oxide—ThS system).
2. Photodynamic therapy, where the fluorescent probe is simultaneously a photosensitizer, generating toxic forms of oxygen only upon irradiation with light of a specific wavelength.
3. Magnetic hyperthermia, where SPIONs serve as an MRI contrast agent and a heat source when exposed to an alternating magnetic field [27].
4. Stimuli-sensitive linkers (pH, GSH), which ensure the release of a therapeutic dose only in the microenvironment of the target, while the diagnostic signal can be obtained from the particle itself before its degradation.

This approach allows administering the particle at a dose sufficient for imaging, and “turning on” the therapeutic effect externally, without increasing systemic toxicity. However, as the authors themselves note, the effectiveness of these strategies in chronic diseases such as AD requires further study, especially concerning repeated use and long-term safety.

Unlike prior scenarios, the fourth—“theranostic pairs”—remains theoretical, lacking AD implementation examples in the current literature. Yet this strategy most comprehensively resolves pharmacokinetic conflicts. It employs two distinct nanoparticle types sharing a common targeting ligand but optimized for divergent functions: diagnostic particles enable rapid accumulation, strong signaling, and swift clearance; therapeutic particles ensure prolonged circulation, controlled release, and sustained therapeutic concentrations. Shared ligands guarantee co-targeting while permitting function-specific optimization—eliminating compromises inherent to single-particle multifunctional designs (Section 4.1). Though absent from analyzed works (which either combine functions or focus unidirectionally), theranostic pairs chart the course for “therapy under diagnostic control.” Although theranostic pairs have not yet been implemented in AD, this concept is already clinically validated in oncology. For example, in patients with intracranial meningioma, ^68^Ga-DOTATATE PET is used for diagnostic imaging, while ^177^Lu-DOTATATE provides targeted radionuclide therapy—both sharing the same ligand (DOTATATE) targeting somatostatin receptor type 2 (SSTR2) [95]. Both agents share the same targeting ligand (DOTATATE) but carry different isotopes optimized for diagnosis (^68^Ga) or therapy (^177^Lu). This paradigm could be adapted for AD by pairing, for instance, a diagnostic nanoparticle labeled with a PET or MRI contrast agent and a therapeutic nanoparticle carrying an anti-aggregation drug, both functionalized with the same Aβ-targeting ligand (e.g., an anti-Aβ antibody or peptide). Such an approach would allow independent optimization of pharmacokinetics and dosing for each function, potentially resolving the dose and pharmacokinetic conflicts discussed in Section 4.

This analysis demonstrates that the “one particle for everything” concept has exhausted its heuristic potential for AD theranostics. Further advancement demands not optimization of existing approaches, but a fundamental paradigm shift. The identified scenarios—preclinical intervention, rapidly progressive forms, theranostic monitoring, and theranostic pairs—represent not merely research directions, but prerequisites for clinical translation. Absent such reconceptualization, AD theranostic systems risk perpetual confinement to “conceptual demonstrations,” never achieving clinical adoption.

## 6. Rethinking the Theranostic Approach: A Roadmap

This analysis demonstrates that current theranostic system development for AD encounters insurmountable conceptual limitations unresolvable through mere particle design complexity. Fundamental revision of design and evaluation principles is imperative, requiring coordinated efforts among developers, clinicians, and scientific journals.

For developers, the evidence base must mandate direct comparisons of integrated systems against separate administration—equivalent diagnostic and therapeutic agent doses given individually. Only such comparisons validate true functional synergy beyond declarative claims. Published studies require quantitative metrics for objective potential assessment: BBB penetration as %ID/g (not fluorescence arbitrary units), diagnostic efficacy via signal-to-noise ratios, therapeutic dosing, and release kinetics. Targeting ligands demand quantitative affinity assessment (Kd) for toxic Aβ oligomers, not merely fibrils. Given AD’s chronicity, priority falls to biodegradable materials enabling repeated dosing without cumulative toxicity or permanent non-degradable particle brain accumulation. CNS nanoparticle elimination mechanisms must constitute mandatory preclinical endpoints—absent these data, theranostic claims remain unsubstantiated. Neurobiologists must transition to models recapitulating sporadic AD chronicity (aging, comorbidities, age-related changes) rather than transgenic mouse rapid amyloidosis.

For clinicians, the priority tasks are the identification of patients with rapidly progressive and monogenic forms of AD (*APP*, *PSEN1*, *PSEN2* mutations), for whom the risk/benefit ratio shifts towards justifying theranostic interventions; validation and implementation of peripheral biomarkers (plasma Aβ42/40, p-tau217, NfL) for detecting the preclinical stage—a niche where theranostic intervention could be effective with single or time-limited application; development of theranostic monitoring protocols using biomarkers and imaging for patient selection, assessing therapy response, and making decisions about treatment continuation. In the long term, registries are necessary to track safety and cognitive outcomes, as preclinical models do not provide answers about cumulative toxicity.

For scientific journals and reviewers, it may be worth considering whether adjustments to editorial policies could further support the development of the field. The sustained interest in AD theranostics stems from a combination of factors, including the genuine biological promise of the approach, as well as institutional and career incentives. The topic’s attractiveness for grant funding and its high publication potential in materials science-oriented journals may sometimes lead to a predominance of proof-of-concept studies that remain at the stage of “conceptual demonstration.” To help the field progress toward clinical translation, a few measures could be considered. For instance, encouraging more rigorous mechanistic justification could be beneficial: demonstrating that a single nanoplatform integrates both diagnostic and therapeutic functions is a valuable first step, but providing quantitative evidence that such integration offers a tangible advantage over the separate administration of diagnostic and therapeutic agents would further strengthen these studies. Additionally, journals might consider requesting key quantitative data needed to assess translational potential (e.g., biodistribution, targeting efficiency, therapeutic index). Finally, creating space for the publication of well-documented negative results could also contribute to a more balanced understanding of the field. Such efforts would help ensure that AD theranostics continues to build on conceptually robust approaches, moving steadily toward clinically meaningful solutions.

## 7. Limitations of Our Analysis

When interpreting the conclusions of this review, it is necessary to consider its genre specificity: we deliberately chose the format of a critical review, rather than a systematic review, since our goal was not an exhaustive catalog of theranostic systems for AD (this task has already been repeatedly addressed in other works), but a conceptual analysis of the very idea of combining diagnostics and therapy. This determined the selectivity of the sample—we focused on representative examples that most vividly illustrate the identified contradictions, and did not strive for completeness of literature coverage. Furthermore, the heterogeneity of the data, due to differences in experimental models, administration protocols, methods of efficacy assessment, and units of measurement, does not allow for a correct quantitative comparison between different studies; however, this was not part of our objectives. Finally, as in any other field, a significant portion of data, especially negative results, remains outside of public access, which may create a distorted picture of the success of certain approaches. Despite the listed limitations, we believe that the proposed conceptual analysis retains its validity, since its conclusions are based not on the frequency of occurrence of certain facts, but on the logic of biological processes and the fundamental requirements for diagnostic and therapeutic agents.

## 8. Conclusions

The conducted critical analysis demonstrates that the concept of theranostics in its current understanding—as “one particle for everything”—faces a number of fundamental and, apparently, insoluble contradictions in relation to AD. The conditions that ensured the success of theranostics in oncology (local solid tumor, EPR effect, BBB absence, acute course, acceptability of high toxicity) are fundamentally absent in AD—a chronic, diffuse neurodegenerative disease with an impregnable BBB and the need for lifelong therapy.

Three fundamental conceptual conflicts were identified. The pharmacokinetic conflict arises from opposing particle behavior requirements: diagnostics demand rapid accumulation and clearance, while therapy necessitates prolonged circulation and sustained release. The dose conflict manifests as diagnostic/therapeutic dose disparities spanning orders of magnitude (13–400-fold or greater), with most studies lacking quantitative dosing data for identical particles; rare instances either employ disparate in vitro/in vivo concentrations or overlook repeated administration’s cumulative effects. The temporal conflict reveals one-time diagnostics’ incompatibility with chronic therapy: long-persisting particles generate irremovable background signals precluding repeat monitoring, while dynamic BBB permeability across disease stages renders any “optimized” particle predictably ineffective elsewhere.

Compounding these conflicts is a methodological trap concerning target selection: most studies target mature Aβ fibril detection/inhibition, despite compelling neurobiological evidence that soluble oligomers represent the most toxic species. Exclusive reliance on ThT efficacy assessment proves methodologically flawed, potentially masking aggregate form redistribution as genuine therapeutic success.

Particle post-function fate remains systematically overlooked. CNS elimination mechanisms go undiscussed, despite non-degradable particle (gold, iron oxide) accumulation in brain parenchyma generating persistent background signals that preclude long-term monitoring and pose chronic neurotoxicity risks. Even pharmacokinetics/safety-focused reviews fail to extend these concerns to the CNS.

Nevertheless, rejecting nanotechnology’s potential entirely would be misguided. Success requires reconceptualizing the paradigm—from “one particle for everything” to flexible, specialized, biologically rational strategies. Most promising directions include: (1) preclinical-phase intervention, where single/time-limited applications delay clinical manifestation; (2) theranostic monitoring, coordinating separate diagnostics and therapy; (3) theranostic pairs—dual particle types sharing targeting ligands but optimized for distinct tasks, resolving pharmacokinetic conflicts without compromise; and (4) external stimuli temporally separating diagnostic/therapeutic functions.

Rather than abandoning theranostics, we must reconceptualize it within AD’s biological realities. The field requires transition from conceptual demonstrations to systematic quantitative analysis, from fibril-centric approaches to toxic oligomer detection, from particle fate neglect to CNS elimination studies, and from single-dose models to chronic therapy paradigms. Principles from successful oncological theranostics—such as shared-ligand theranostic pairs and on-demand therapy activation—can be adapted for AD, provided the unique challenges of diffuse pathology, the BBB, and chronic disease course are taken into account. Only such paradigm shift can transform theranostics from elegant chemical concepts into clinically viable tools for early AD and effective treatment.

## Figures and Tables

**Table 1:** Characteristics of nanotheranostic systems for AD.

Nanoparticle Type	Size, nm	Zeta Potential, mV	Therapeutic Agent	Imaging Method	Target	Animal Model	Cell Model	Administration Route	Key Results	Reference
Polymeric and nanocomposites										
NaYF4:Yb/Er/Tm@DSPE-PEG@HQC NPs (UCHQs)	114	31.2	HQC (Cu^2+^ chelator)	Luminescence	Aβ (via Cu^2+^)	Transgenic AD model—APPsw/PSEN1 mice	PC-12 cell	i.c., 0.4 mg/kg, 25 μL per mice, single dose; i.c., 200 μg/mL per zebrafish embryos, single dose; In vitro: 0–500 μg/mL (cytotoxicity); 50 μmol/L (apoptosis).	Detect and capture Cu^2+^; inhibit Aβ aggregation.	Cui et al., 2016 [69]
Chitosan-hyaluronic acid NPs (CHG)	110.4 ± 15.6	32.0 ± 3.1	Particle itself (Aβ aggregation inhibitor)	Fluorescence	Aβ oligomers and fibrils	Transgenic AD model—*C. elegans*	SHSY-5Y cells	*C. elegans*: 30 μg/mL (imaging, fixed nematodes), 300 μg/mL (therapy, ingested with food, 72 h); in vitro: CHG NPs 7 μg/mL (probing), 90–720 μg/mL (inhibition, MTT).	Selectively bind to Aβ aggregates; inhibit fibrillogenesis; fluorescence enhances upon binding.	Wang et al., 2021 [68]
Polymeric nanocore with Magnevist^®^ (TNV)	239 ± 4.1	11.9 ± 0.5	Cyclophosphamide, anti-amyloid antibody Putrescine modified F(ab’)2 fragment	MRI, SPECT	Aβ, inflammation	Cerebral amyloid angiopathy model—B6SJLF1/J mice	hCMEC/D3 cells, BBMVEC cells	i.v., single dose: ^125^I-TNVs 100 μCi (PK/biodistribution) or 500 μCi (SPECT/CT), TNVs 200 μL with 2 mM Gd (MRI). In vitro: TNV 30 μg/mL (cells uptake); TNV 1.74 mg/mL (cytokine inhibition in cells).	Target cerebrovascular amyloid; reduce pro-inflammatory cytokine production.	Agyare et al., 2014 [70]
Chitosan-based NPs with Gd-DTPA (gadolinium-diethylene triamine pentaacetic acid)	145–158	4.5–7.7	Curcumin or dexamethasone + IgG4.1 antibody	MRI, SPECT	Aβ, inflammation	Transgenic AD model—Tg2576 transgenic mice; B6/SJL mice	hCMEC/D3 cells	i.v. (femoral vein, single dose: 100 µCi ^125^I-nanovehicles (PK), 17 mg (MRI), 500 µCi (SPECT); external carotid artery (single dose: 45 mg nanovehicles).	Accumulate in brain vasculature, target amyloid; provide contrast and reduce inflammation.	Jaruszewski et al., 2014 [71]
DSPE-PEG-SPIO with curcumin (SDP@Cur-CRT/QSH)	180	~–19	Curcumin	MRI	Aβ, inflammation (NLRP3)	Transgenic AD model—PP/PS1 mice	n/a	i.v. tail vein, 25 mg/kg, every 4 days for 3 months; i.v. tail vein 200 µL Fe/kg, single dose, (MRI); In vitro: 25–200 µg/mL for cells cytotoxicity; 100 µg/mL for uptake.	Visualize plaques, reduce their burden; improve cognitive function.	Ruan et al., 2022 [58]
CeNC/IONC/MSN-T807 nanocomposite	131.6	9.32	Methylene blue (MB) (tau aggregation inhibitor), CeNC (antioxidant and suppressor tau hyperphosphorylation)	MRI/PET bimodal imaging (IONCs and 68Ga)	Hyperphosphorylated tau, oxidative stress	Intrahippocampal okadaic acid injection—Sprague-Dawley rats	SH-SY5Y cells	i.h. (unilateral hippocampus) 10 μL (MB 3.6 mg/mL equivalent), single dose (therapy); i.h. 2 μL (20 mg/mL) for MRI; in vitro (cells): MB 2.25 μg/mL, nanocomposite 4–6.25 μg/mL.	Reduces tau hyperphosphorylation, oxidative stress, and neuronal apoptosis; improves memory in rats.	Chen et al., 2018 [64]
IR780-Mn@TA-TPL NPs (DSPE-PEG-based)	116	–16.5	Tannic acid (antioxidant)	MRI (Mn^2+^ ions), fluorescence (IR780)	Oxidative stress, tau	Intracerebral aluminum oxide injection—Sprague-Dawley	PC12 and BV2 cells	i.v., single dose (imaging); i.h. single dose (MRI); in vitro: 50 µg/mL for cellular uptake.	Reduce ROS and tau hyperphosphorylation; restore memory.	Gu et al., 2024 [72]
PHEMA-RA-PCB-CPP/SPION/siSOX9	100	2.6 (pH 7.4)—15.4 (pH 3.5)	siSOX9, retinoic acid	MRI	NSCs	Transgenic AD model—2 × Tg-AD mice	n/a	Transplantation of NSCs pre-treated with NPs (into brain), dose not specified, single dose (for therapy); MRI at day 1 and day 35 post-transplantation.	Control NSC differentiation into neurons; suppress SOX9; improve memory.	Zhang et al., 2016 [73]
Lipid nanoparticles and exosomes										
Liposomal immunoglobulins (mf-LIPs) with three ligands on the surface	149 ± 25	–3.01 to –3.71	Curcumin-lipid ligand (TREG), ligand for action on transferrin and low-density lipoprotein receptors of the BBB (TfR-Mab), ApoE	Biofluorescence	Aβ, BBB receptors	FVB mice (wild-type)	In vitro BBB model—hCMEC/D3 cells	i.v. (tail vein) 0.05 mg/mouse, single dose; 200 nmol lipid/well (permeation); 200 nmol/10^6^ cells (uptake); 40 mM (thioflavin-T aggregation assay).	Inhibit Aβ aggregation in vitro; cross BBB; stable.	Papadia et al., 2017 [74]
Modified neutrophil-derived exosomes (MP@Cur-MExo)	120	–20.6	Curcumin, SPION	MRI/IVIS (fluorescence) bimodal imaging	Aβ, mitochondria	Transgenic AD model—APP/PS1 mice	n/a	i.v. injection, dose 1 mg/mL per mice for MRI; in vitro: 10^5^ exosomes/well (cells).	Accumulate in inflamed brain areas; protect neurons; improve cognitive function.	Zhang et al., 2024 [75]
Carbon dots (CDs)										
Nitrogen-doped carbon dots (N-CD)	2.2	n/a	Particle itself	Fluorescence	Aβ	n/a	n/a	In vitro only: N-CD 0.75–6.0 mg/mL + Aβ1-42 (100 μM), 24 h for cells.	Reduce Aβ content 5-fold; inhibit Aβ1-42 self-assembly.	Liu et al., 2023 [76]
EGCG-derived polymer dots (E-CPDs)	1.9 ± 0.4	–17.5	EGCG	Fluorescence	Aβ	Transgenic AD model—*C. elegans* (CL2006)	SH-SY5Y cells	addition to nematode growth medium (80 µg/mL).	Inhibit fibrillization and disaggregate Aβ fibrils; extend nematode lifespan.	Lin et al., 2023 [77]
Cerium-containing carbon dots (CCP-CD)	n/a	n/a	Curcumin, Ce^3+^	Fluorescence (p-PD)	Aβ, oxidative stress	Transgenic AD model—*C. elegans*	n/a	1–10 μg/mL (ROS).	Reduce ROS and inflammation; inhibit Aβ fibrillization; extend nematode lifespan.	Wei et al., 2025 [78]
Quercetin and p-PD -derived carbon dots (R-CD-75)	3.66 ± 0.03	–12.38	Quercetin, CDs	Fluorescence	Aβ, ROS	Transgenic AD model—*C. elegans* (CL2006)	SH-SY5Y cells	in vitro: 2–50 μg/mL.	Inhibit aggregation and disaggregate Aβ fibrils; reduce ROS; extend nematode lifespan by 50%.	Wei et al., 2024 [79]
HSA-BFP@CDs nanocomposite	37.8	n/a	HSA-BFP, CDs	Fluorescence	Aβ, ROS	Transgenic AD model—*C. elegans* (CL2006)	SH-SY5Y cells	*C. elegans*: added to nematode growth medium, 30 µg/mL for 4 h (imaging), 100 µg/mL for 72 h (deposition, ROS, distribution, lifespan), 100 µg/mL; in vitro: 10–100 µg/mL (cell viability, ROS).	Detect plaques; reduce ROS and Aβ cytotoxicity.	Wang et al., 2022 [80]
Gold nanorods										
AuNRs with POM and Aβ15-20 (AuP)	n/a	n/a	POM, Aβ15-20 peptide	Fluorescence	Aβ	S4880202 mice	PC12 cells	i.v. tail vein, single dose 100 µL AuP (120 µg/mL Au), analyzed at 6 h (BBB permeability). In vitro: 50 µM Aβ + 0.3 nM AuP (ThS Staining).	Disrupt Aβ fibrils under NIR; protect cells; cross BBB.	Li et al., 2017 [29]
GNRs-APH-scFv (GAS)	n/a	15.8 (GAS)	scFv 12B4, APH ST0779	NIR fluorescence	Aβ	Transgenic AD model—*C. elegans* (CL4176)	n/a	Via food—40 µM per nematode growth medium plate.	Detect Aβ aggregates; under NIR disrupt fibrils; delay paralysis.	Liu et al., 2019 [81]
Magnetic nanoparticles										
SPION with PH-1/PH-2	12.6 ± 0.7	n/a	SPION	MRI, fluorescence	Aβ	Transgenic AD model—APP/PS1 mice	n/a	i.v. 0.2 mmol Fe/kg, single dose (imaging); in vitro: concentrations 1–100 ng/mL (cytotoxicity, disaggregation of Aβ1-42).	Bind to Aβ; block aggregation; protect SH-SY5Y cells.	Cai et al., 2020 [82]
W20/XD4-SPION	11.5 ± 1.8	–15	SPION + W20	MRI	Aβ oligomers	Transgenic AD model—APP/PS1 mice	BV-2 cells	i.v. tail vein, 200 μmol Fe/kg, single dose (distribution, MRI).	Target Aβ oligomers; enhance microglial phagocytosis; differentiate transgenic mice.	Liu et al., 2020 [63]
Congo Red/Rutin-MNPs based on USPION, DSPE-PEG and Oleic acid	35–45	n/a	Rutin	MRI	Aβ, oxidative stress	Transgenic AD model—APP/PS1 mice	SH-SY5Y cells	i.v. (co-injected with mannitol), dose not specified, single dose.	Visualize plaques; H_2_O_2_-responsive release; reduce ROS; improve memory.	Hu et al., 2015 [83]
Miscellaneous										
Charged dye molecule DBA-SLOH.	n/a	n/a	Particle itself	Fluorescence	Aβ	Transgenic AD model—APP/PS1 mice	n/a	i.v. (tail vein) 5 mg/kg, single dose (100 μL); in vitro: 1–50 μM (cytotoxicity), 100 μM (aggregation inhibition).	High Aβ affinity, BBB-permeable. Inhibits Aβ aggregation.	Li et al., 2016 [84]
Tryptophan Nps (TNPs)	82–121	n/a	Tryptophan	Fluorescence	Aβ42, phenylalanine-phenylalanine -dipeptides	Streptozotocin injection—Sprague-Dawley rats	SH-SY5Y cells	i.v. 5 mg/kg every third day for 26 days (therapy); in vitro: TNPs 100 μg/mL.	Inhibit Aβ aggregation; disrupt fibrils; improve memory in rats.	Sharma et al., 2022 [85]
^11^C-pyrimidine radioligands	n/a	n/a	Particle itself	PET	σ1 receptor	Transgenic AD model—5xFAD mice; NHP	n/a	i.v. mice tail vein 200 µL (PET); single dose; NHP—i.v. NHP 5.1 mCi (PET/MRI); single dose.	Bind to σ1R; sensitive to σ1R downregulation in pathology.	Bai et al., 2025 [86]

Abbreviations: BBB—blood–brain barrier; ROS—reactive oxygen species; NSCs—neural stem cells; SPECT—single-photon emission computed tomography; PET—positron emission tomography; NIR—near-infrared; hCMEC/D3—human brain microvascular endothelial cells; BBMVEC—bovine brain microvascular endothelial cells; SPIO/SPION—superparamagnetic iron oxide (nanoparticles); USPION—ultrasmall superparamagnetic iron oxide nanoparticles; DSPE-PEG—1,2-dioleoyl-sn-glycero-3-phosphoethanolamine-n-[poly(ethylene glycol)]; NLRP3—NOD-like receptor family pyrin domain containing 3 inflammasome; SOX9—SRY-box transcription factor 9; HQC—8-hydroxyquinoline-2-carboxylic acid; p-PD—p-phenylenediamine; CDs—carbon dots; HSA-BFP—triple-functionalized human serum albumin; EGCG—epigallocatechin gallate; POM—polyoxometalates; W20—oligomer-specific scFv antibody; XD4—class A scavenger receptor (SR-A) activator; DBA-SLOH—(E)-4-(4-(dibutylamino)styryl)-1-(2-hydroxyethyl)quinolin-1-ium chloride; n/a—not available/data not provided.

## Data Availability

No new data were created or analyzed in this study. Data sharing is not applicable to this article.

## Abbreviations

The following abbreviations are used in this manuscript:

AD	Alzheimer’s disease
APP	Amyloid precursor protein
BBB	Blood–Brain Barrier
CDs	Carbon dots
CNS	Central nervous system
CSF	Cerebrospinal Fluid
EPR	Enhanced Permeability and Retention
FDA	Food and Drug Administration
MRI	Magnetic Resonance Imaging
NIR	Near infrared
NPs	Nanoparticles
PET	Positron Emission Tomography
QDs	Quantum Dots
ROS	Reactive Oxygen Species
SPECT	Single-Photon Emission Computed Tomography
SPIONs	Superparamagnetic iron oxide nanoparticles
ThS	Thioflavin-S
ThT	Thioflavin-T
